# Frequency of Acute Hepatitis Following Acute Paraphenylene Diamine Intoxication

**DOI:** 10.7759/cureus.1186

**Published:** 2017-04-21

**Authors:** Rizwan Ishtiaq, Sadaf Shafiq, Ali Imran, Qazi Masroor Ali, Raheel Khan, Hassan Tariq, Daniyal Ishtiaq

**Affiliations:** 1 General Medicine, Quaid-e-Azam Medical College, Bahawalpur, Pakistan; 2 Department of Pathology, Quaid-e-Azam Medical College, Bahawalpur, Pakistan; 3 Assistant Professor of Medicine, Quaid-e-Azam Medical College, Bahawalpur, Pakistan; 4 Professor of Medicine, Quaid-e-Azam Medical College, Bahawalpur, Pakistan; 5 Psychiatry, Quaid-e-Azam Medical College, Bahawalpur, Pakistan; 6 General Medicine, Rawalpindi Medical College, Rawalpindi, Pakistan

**Keywords:** paraphenylene diamine, intoxication, hepatitis, renal failure

## Abstract

**Introduction:**

Paraphenylene diamine (PPD) ingestion is manifesting as one of the more common ways of committing suicide in Southern Punjab, Pakistan, especially Bahawalpur. PPD is an ingredient of a compound commonly known “Kala Pathar” which means “Black Stone” in Urdu. It is readily available in the market at low cost and is used to dye hair and fur. Its intoxication inhibits cellular oxidation and affects the muscles causing rhabdomyolysis. This leads to myoglobinuria followed by renal failure and edema of face and throat resulting in respiratory difficulty. Very little is known about the impact of PPD intoxication on liver tissue.

**Objective:**

The purpose of the study was to find out the frequency of acute hepatitis following PPD intoxication.

**Methods:**

We reviewed the medical records of 109 patients with PPD intoxication admitted to Medical Unit-2, Bahawalpur Victoria Hospital from January 1, 2015, to June 30, 2015, in a descriptive, cross-sectional study. We noted the frequency of acute hepatitis and other complications, and we recorded the demographic features, clinical features, and outcomes of these patients.

**Results:**

Our study included 32 men (29%) and 77 women (71%). The mean age was 22 ± 3.4 years, and most patients were young women aged 15 to 24 years. Suicidal ingestion was the leading cause of admission for 101 patients (93%). The most common clinical presentation was cervicofacial edema (95%), throat pain (88%), dysphonia (95%), cola-colored urine (100%), and oliguria (95%). Rhabdomyolysis (86%), acute hepatitis (51%), and acute renal failure (63%) were the most common clinical conditions following poisoning. Overall mortality was noted in 39 patients (36%) while all other patients achieved complete clinical recovery (64%). In patients with mortality, 20 of 39 (51%) developed acute hepatitis. Most patients (95%) in our study underwent tracheostomy.

**Conclusion:**

The frequency of acute hepatitis in PPD intoxication is high in this population, especially in young women. Measures need to be instituted regarding the management of acute hepatitis in PPD intoxication to improve patient outcomes. Workups in patients with PPD poisoning should include regular monitoring of aspartate aminotransferase and alanine aminotransferase to observe any damages to the liver so that acute hepatitis can be managed in a timely fashion.

## Introduction

Suicide is a major health problem not only in developing countries but also in developed nations of the world; suicide accounts for more than one million fatalities worldwide [1]. In developing countries like Pakistan where illiteracy is high, and poverty is common, intoxication with paraphenylene diamine (PPD) has become a common method of self-harm because it is cheap and readily available in the market. In 1990, PPD intoxication was the leading cause of poisoning in Morocco [2]. PPD is an active ingredient in a compound commonly known as “Kala Pathar,” which means “Black Stone” in Urdu. It is available in the market in the form of crystals which are ground and used to dye hair and furs when mixed with henna. The most common clinical presentations after PPD intoxication include cervicofacial edema, rhabdomyolysis causing myoglobinuria, cola-colored urine, oliguria, and renal failure caused by acute tubular necrosis [3]. Common laboratory test result abnormalities observed include increased lactate dehydrogenase levels, increased creatinine phosphokinase (CPK) levels, and hyperkalemia. To date, no effective antidote for this chemical has been discovered. It is nondialyzable, and all patients are managed conservatively [4]. Management includes early tracheostomy for cervicofacial edema and intravenous (IV) fluids with aggressive diuresis and urine alkalization for renal failure. Patients are usually treated for kidney failure and respiratory compromise but very little effort is made for acute hepatitis due to PPD intoxication. Very little is known about PPD’s impact on liver tissue. To our knowledge, no studies have been published on the effects of PPD on liver tissue and hepatitis. We present the first study to emphasize the significance of PPD in the development of hepatitis. Our study indicates acute hepatitis is one of the leading causes of mortality along with renal failure, and measures need to be instituted regarding the management of acute hepatitis in cases of PPD intoxication to improve the outcome of patients and decrease mortality.

## Materials and methods

This descriptive, cross-sectional study took place in Medical Unit 2 of Bahawal Victoria Hospital, Bahawalpur, which is a tertiary care hospital in Southern Punjab, Pakistan. We reviewed the medical records of 109 patients with PPD intoxication who were admitted to the Medical Unit 2 of Bahawal Victoria Hospital from January 1, 2015, to June 30, 2015. This study was approved by the Institutional Review Board of Quaid-e-Azam Medical College, Bahawalpur. Patients or their guardians provided informed consent, and patient information remained confidential. A predesigned pro forma was used to collect information regarding social class, social conflict, the reason for intoxication, location (i.e., rural or urban), the duration between ingestion and presentation in the hospital, and outcome. Clinical symptoms and laboratory test results were gathered from patients’ medical records.

Statistical analysis was performed using Statistical Package for Social Sciences version 23.0 (IBM Corp., Armonk, NY).

Treatment was started immediately using oxygen inhalation, antihistamines, parental steroids, aggressive IV fluid therapy with diuresis, and alkalization of urine. Patients with cervicofacial edema received tracheostomies to keep airways patent and to prevent respiratory compromise. Patient input and output were monitored carefully, and charts were maintained. Tests for renal function, liver function, serum electrolyte levels, and serum CPK levels were regularly ordered.

Given the lack of an antidote and guidelines about the management of hepatitis in PPD intoxication, our management for hepatitis was supportive (i.e., IV fluid therapy and antibiotics to prevent sepsis). If the timing of ingestion was recent, gastric lavage was performed in some patients once their airways were secured. All patients were regularly monitored for transaminase levels, prothrombin time, bilirubin levels, and hypoglycemia. In patients with clinical recovery, attendants and patients were counseled, and the patient was referred for psychiatric evaluation.

## Results

**Figure 1:**
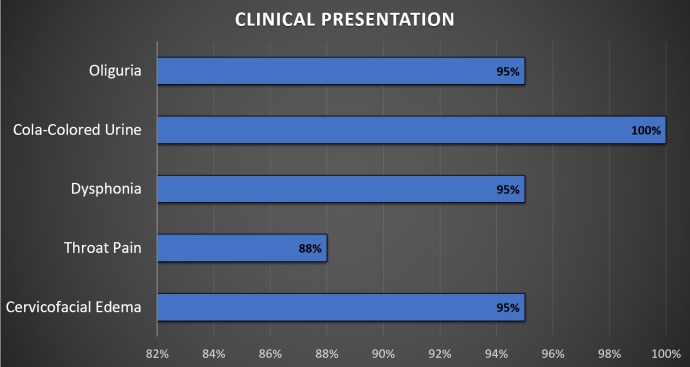
Clinical presentation after paraphenylene diamine (PPD) intoxication

Our study included 32 men (29%) and 77 women (71%). The mean age was 22 ± 3.4 years, and most patients were young women aged 15 to 24 years. Seventy-eight patients (72%) belonged to a low socio-economic class, and 31 patients (28%) belonged to middle-class families. Suicidal ingestion was the leading cause of admission for 101 patients (93%) due to personal conflicts at home or poverty. Eight patients (7%) came to the hospital because of accidental ingestion of Kala Pathar. These eight patients were all children ranging in age from nine to 12 years. Eighty-one patients (74%) came from rural areas, and 28 patients (26%) came from urban settings. Figure 1 presents the most common clinical presentations.

**Figure 2:**
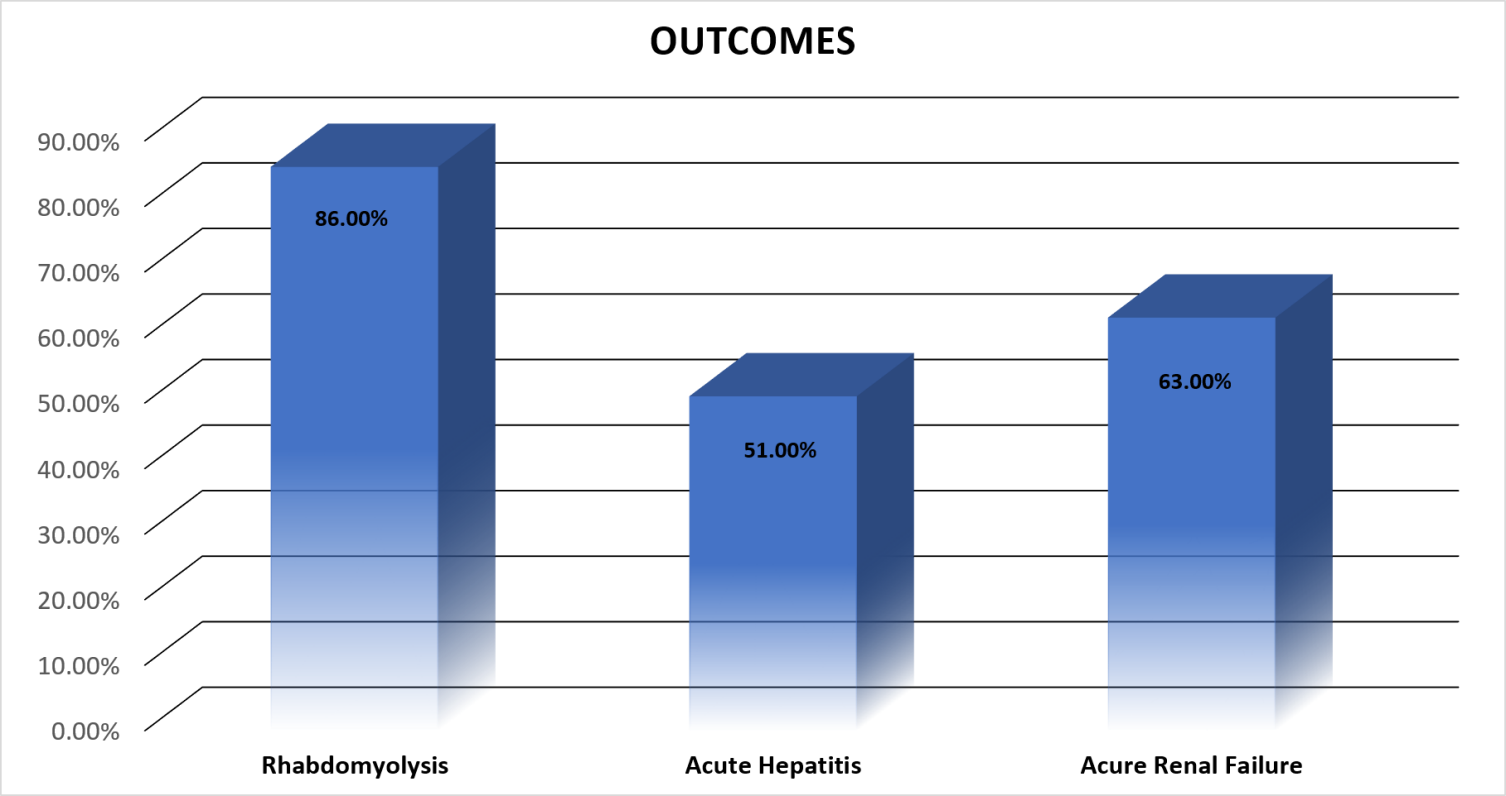
Outcomes after paraphenylene diamine (PPD) intoxication

Figure 2 presents clinical presentations following poisoning. The mean ± standard deviations of AST levels and ALT levels were 2956 ± 3024 and 1045 ± 1137, respectively.

Overall, mortality was noted in 39 patients (36%); all remaining patients achieved complete clinical recovery (64%). In patients with mortality, 12 of 39 (30%) were men, and 27 of 39 (70%) were women. Twenty of 39 patients (51%) developed acute hepatitis. Thirty-seven of 39 (95%) developed renal failure. Of the two patients who did not have renal failure, one patient developed acute hepatitis. Fifty-six of 109 (51%) patients developed acute hepatitis, and mortality was seen in 20 of 56 (35.7%) patients. Ninety-five percent of the patients in our study underwent tracheostomy.

## Discussion

PPD is a cheap compound available over the counter that is formulated to enhance the colors used to dye hair and fur. In 1924, the first case of PPD intoxication was documented when a barber accidently developed toxicity while handling the dye [5]. Women are found more vulnerable to the misuse of PPD not only because of their use in enhancing hair colors but also because women are typically under more social pressure than men, especially in developing countries like Pakistan [2]. The chemical formula for this compound is C6H4 (NH2)2 [6]. It is a derivative of a white substance that turns black on oxidation called paraphenylaniline, which is soluble in hydrogen peroxide but not in water. With the help of the cytochrome P450 peroxidase system, PPD is metabolized into a reactive agent called benzoquinone diamine. Further oxidation leads to the formation of Brandowaski’s base, which is reported to cause anaphylaxis [7]. This chemical is extremely toxic to living tissues; it blocks cellular oxidation mechanisms and can cause death within 24 hours due to respiratory failure [8]. Its misuse is well known to cause insults to the patency of airways in the neck and the renal tissue (ultimately leading to renal failure). In our clinical setting, we noted that PPD has potential to cause damage to the hepatocytes and leads to fulminant hepatic failure. Most of the mortality cases in our study had a documented liver damage after PPD ingestion due to increased levels of aspartate transaminase and alanine transaminase.

PPD is available in crystal, liquid, or powder formations. While the literature currently lacks reported mortality rates for each form, one study indicates the crystal formation carries the highest mortality rate [9]. The minimum lethal dose is reported to be 7 to 10 grams, although intake of 3 grams is sufficient to cause vital damage [10]. The median toxic dose (LD50) of PPD is 0.028 mg/L [11]. This indicates PPD’s effects are dose-related. Cervicofacial edema, cola-colored urine, and respiratory distress are the earliest signs to appear. Late complications include rhabdomyolysis, hepatitis, acute renal failure, acute tubular necrosis, arrhythmias, and methemoglobinemia [12]. In the absence of proper history and proper laboratory investigations, the triad of cervicofacial edema, cola-colored urine and oliguria can be confirming evidence of PPD poisoning [11]. One study documented alveolar rupture due to air trapping from laryngeal edema [13]. Predictors of mortality due to PPD intoxication include the dose of toxin ingested, hyperkalemia, hyperphosphatemia, and hypercalcemia [14]. Hyperkalemia is important because it can lead to arrhythmias which can be potentiated by the PPD toxin itself and result in sudden cardiac death. Cervicofacial edema is the gravest and most common sign of PPD intoxication, and it can result in fatal respiratory distress if not addressed urgently. Any sign of neck swelling after PPD intoxication is an indication for an urgent tracheostomy; the swelling neck will collapse the patient’s airway if an immediate tracheostomy is not performed. In emergency settings, tracheostomy due to PPD intoxication is becoming one of the most common reasons for tracheostomy in southern Asia [15].

The management of PPD intoxication is mainly dependent on keeping the patency of airways and preventing acute renal failure. Currently, there is no available antidote to neutralize the toxic effects of PPD. Treating hepatitis in PPD intoxication is mainly supportive. In our study, we treated our patients with IV fluids and antibiotics for the prevention of sepsis. Little information is available in the literature regarding the specific treatment of hepatitis in PPD patients. According to our study, liver failure was the most masked presentation due to the absence of any physical findings. According to one study, consumption of as little as 25 mL of liquid PPD toxin can lead to hepatitis [16]. Further studies may elucidate the predictive value of hepatic enzymes like ALT and AST. Determining the predictive value of hepatic enzymes will not only help guide the management of PPD intoxication but may also decrease PPD-related mortality.

## Conclusions

Diagnosing PPD intoxication requires a high degree of suspicion, especially when the triad of cervicofacial edema, cola-colored urine, and oliguria is present. Acute hepatitis is a common complication following PPD ingestion. Clinicians and intensivists should be aware of PPD’s adverse effects on the liver and the potential outcomes so that a proper management plan and potentially preventive measures can be mapped out. Primary care physicians, intensivists, and nephrologists must take into consideration the hepatic damage caused by the PPD toxin when examining PPD-intoxicated patients. Aspartate transaminase and alanine transaminase levels should be made a part of the regular workup of PPD-intoxicated patients, and those levels should be monitored daily. All patients with suicidal intent should be referred to a psychiatrist for proper evaluation and counseling.

While treatment guidelines are being explored and updated, further research should be conducted to find a specific antidote with a specific focus on liver pathology. Concurrently, authorities and policy makers should take strict actions to regulate the currently open and easy public access to such chemicals.

## References

[1] World Health (2017). World Health Organization Suicide Data. Suicide.

[2] Benslama A, Moutaouakkil S, Mjahed K (1998). Intermediary syndrome in acute malathion poisoning. (Article in French). Presse Medicale.

[3] Akbar MA, Khaliq SA, Malik NA (2010). Kala Pathar (paraphenylene diamin) intoxication; a study at Nishtar Hospital Multan. Nishtar Med J.

[4] Kondle R, Pathapati RM, Saginela SK (2012). Clinical profile and outcomes of hair dye poisoning in a teaching hospital in Nellore. ISRN Emerg Med.

[5] Nott HW (1924). Systemic poisoning by hair dye. Br Med J.

[6] Khan H, Khan N, Khan N (2015). Clinical presentation and outcome of patients with paraphenylenediamine (kala-pathar) poisoning. Gomal J Med Sci.

[7] Sakuntala P, Khan PM, Sudarsi B (2015). Clinical profile and complications of hair dye poisoning. Int J Sci Res Pub.

[8] Suliman SM, Fadlalla M, Nasr ME (1995). Poisoning with hair-dye containing paraphenylene diamine: ten years experience. Saudi J Kidney Dis.

[9] Abdelraheem MB, Elbushra M, Ali ET (2012). Filicide and suicide in a family by paraphenylene diamine poisoning: a mother who committed suicide and poisoned her four children of which one died. Toxicol Ind Health.

[10] Jain PK, Agarwal N, Kumar P (2011). Hair dye poisoning in Bundelkhand region (Prospective Analysis of Hair Dye Poisoning Cases presented in Department of Medicine, MLB Medical College, Jhansi). J Assoc Physicians India.

[11] Smiley RA (2000). Phenylene- and Toluenediamines. Ullmann's Encyclopedia of Industrial Chemistry.

[12] White JM, Kullavanijaya P, Duangdeeden I (2006). p‐Phenylenediamine allergy: the role of Bandrowski's base. Clin Exp Allergy.

[13] Chrispal A, Begum A, Ramya I (2010). Hair dye poisoning–an emerging problem in the tropics: an experience from a tertiary care hospital in South India. Trop Doct.

[14] Senthilkumaran S, Thirumalaikolundusubramanian P (2015). Acute hair dye poisoning: lurking dangers. J Mahatma Gandhi Inst Med Sci.

[15] Ishaque S, Haq A, Jurair H (2016). Kaala pathar (paraphenylene diamine) poisoning and angioedema in a child: an unusual encounter. J Clin Toxicol.

[16] Hou FQ, Lin XH, Yu YY (2009). Severe liver injury induced by repeated use of hair dye. Chin Med J (Engl).

